# BTEX biodegradation by *Bacillus amyloliquefaciens* subsp. *plantarum* W1 and its proposed BTEX biodegradation pathways

**DOI:** 10.1038/s41598-020-74570-3

**Published:** 2020-10-15

**Authors:** Akanit Wongbunmak, Sansanee Khiawjan, Manop Suphantharika, Thunyarat Pongtharangkul

**Affiliations:** Department of Biotechnology, Faculty of Science, Mahidol University, Bangkok, 10400 Thailand

**Keywords:** Biotechnology, Microbiology

## Abstract

Benzene, toluene, ethylbenzene and (*p-*, *m-* and *o-*) xylene (BTEX) are classified as main pollutants by several environmental protection agencies. In this study, a non-pathogenic, Gram-positive rod-shape bacterium with an ability to degrade all six BTEX compounds, employed as an individual substrate or as a mixture, was isolated. The bacterial isolate was identified as *Bacillus amyloliquefaciens* subsp. *plantarum* strain W1. An overall BTEX biodegradation (as individual substrates) by strain W1 could be ranked as: toluene > benzene, ethylbenzene, *p-*xylene > *m-*xylene > *o-*xylene. When presented in a BTEX mixture, *m-*xylene and *o-*xylene biodegradation was slightly improved suggesting an induction effect by other BTEX components. BTEX biodegradation pathways of strain W1 were proposed based on analyses of its metabolic intermediates identified by LC–MS/MS. Detected activity of several putative monooxygenases and dioxygenases suggested the versatility of strain W1. Thus far, this is the first report of biodegradation pathways for all of the six BTEX compounds by a unique bacterium of the genus *Bacillus*. Moreover, *B. amyloliquefaciens* subsp. *plantarum* W1 could be a good candidate for an in situ bioremediation considering its Generally Recognized as Safe (GRAS) status and a possibility to serve as a plant growth-promoting rhizobacterium (PGPR).

## Introduction

BTEX, a group of volatile organic compounds (VOCs) composed of benzene, toluene, ethylbenzene and three isomers of xylene (*p-*xylene, *m-*xylene and *o-*xylene), are classified as main pollutants by the US Environmental Protection Agency. The widespread contamination of BTEX compounds in an environment is frequently associated with oil spills, discharges from petroleum industries and engine combustion^1–3^. BTEX components are the most water soluble and mobile fraction of crude oil and several petroleum products^4^.

Once BTEX contaminate an environment, they can easily enter a human body through several routes including ingestion, inhalation and skin exposure^5^. Exposure of an individual BTEX chemical could lead to neurological impairment and long-term exposure to benzene significantly increased the occurrence of anemia, excessive bleeding and leukemia^6^. Kidney and inner ear damages were found to be associated with long-term exposure to ethylbenzene in animals^7^**.** Therefore, the International Agency for Research on Cancer has classified ethylbenzene as a possible human carcinogen^8^.

There are several strategies for BTEX removal from soil and groundwater including physical treatment, chemical treatment and biological treatment or biodegradation. Biodegradation, widely regarded as a cost-effective and environmentally friendly strategy, uses the biological process or microorganisms to convert toxic compounds into less toxic compounds^9^. During the biodegradation process, BTEX can be used as carbon sources for microbial growth, the toxic pollutants are therefore completely mineralized in the process^10^. Naturally, BTEX are gradually degraded by indigenous bacteria on site^11^. In many cases, however, the indigenous bacteria could not effectively remove BTEX either because of an unfavorable condition for BTEX biodegradation or a lack of effective BTEX degraders in the contaminated site^12,13^. Thus, an isolation and characterization of BTEX-degrading bacteria from their natural habitat is an important key for successful biodegradation.

During the past decade, many researchers have isolated and identified various microbial species with the ability to degrade BTEX compounds from the environment. Among them, genus *Pseudomonas* was the most frequently reported, for example, *Pseudomonas putida*^14^, *Pseudomonas fluorescens*^15^, *Pseudomonas* sp. BTEX-30^16^ and *Pseudomonas stutzeri*^17^. While other bacteria such as *Rhodococcus rhodochrous*^18^, *Acinetobacter baumannii*^19^ and *Microbacterium esteraromaticum* SBS1-7^20^ were reported specifically for their BTEX degradation, the genus *Bacillus* was the least studied regarding BTEX biodegradation. Most *Bacillus* were, however, reported as a potent crude oil degrader^21^. Although *Bacillus pumilus* MVSV3 was reported to completely degrade 150 mg/L (or ppm) of BTEX within 48 h^22^, *B. pumilus* has been reported to cause a cutaneous infection similar to an anthrax lesion^23^. Therefore, the strain may not be suitable for an application in an open system*.* Moreover, catabolic pathways for BTEX biodegradation were well characterized in Gram negative bacteria but not in Gram positive bacteria, especially in the genera *Bacillus*.

This study aimed to evaluate the potential of a non-pathogenic bacterium *Bacillus amyloliquefaciens* strain W1 for bioremediation of BTEX. Strain W1 was, therefore, evaluated for its ability to utilize BTEX compounds (as an individual substrate or a mixture) in various conditions (liquid medium and soil slurry system). In addition, the metabolites formed during the biodegradation of each BTEX compound were identified by LC–MS/MS and possible BTEX biodegradation pathways employed by the strain were reported.

## Results and discussion

### Isolation and identification of BTEX-degrading *B. amyloliquefaciens* W1 from petroleum waste

After a month-long enrichment, a total of 29 bacterial isolates with different colony morphologies were obtained. Among them, isolate W1 with a white and an irregular shape colony exhibited the ability to grow in mineral salt medium (MM) supplemented with 600 mg/L (ppm) of BTEX mixture within 3 days. Based on the sequence similarity of 16s rDNA (deposited in the GenBank under an accession number MN966853), isolate W1 was closely related to *Bacillus amyloliquefaciens* (99% similarity).

*B. amyloliquefaciens* is a Gram-positive, aerobic, endospore-forming bacterium widely used in the industrial production of *α-*amylase and protease. This species can be divided into 2 subspecies including (1) *B. amyloliquefaciens* subsp. *amyloliquefaciens* with *B. amyloliquefaciens* DSM7^T^ as a type strain and (2) *B. amyloliquefaciens* subsp. *plantarum* with *B. amyloliquefaciens* FZB42^T^ as a type strain. The members of the *B. amyloliquefaciens* subsp. *plantarum* were reported to harbor an *amy*E-like gene instead of an *amy*A-like gene found in *B. amyloliquefaciens* subsp. *amyloliquefaciens*^24^. The presence of *amy*A or *amy*E gene in a genomic DNA of isolate W1 was confirmed by a polymerase chain reaction (PCR) using primers specific for each gene (Table S1, supplementary material online). The obtained *amy*E-like gene fragment (1980 bp) confirmed that isolate W1 should be classified as *B. amyloliquefaciens* subsp. *plantarum* (Fig. S1).

It should be noted that *B. amyloliquefaciens* subsp. *plantarum* was frequently reported as a plant growth promoting rhizobacterium (PGPR) with the ability to colonize the roots of plants as well as to produce plant hormones such as indole-3-acetic acid (IAA)^25–27^. Therefore, isolate W1 should be regarded as safe for applications in an open environment. Later on, the strain should be evaluated for its PGPR properties (e.g. production of IAA/siderophore/antimicrobial agents and phosphate solubilization) in order to extend its application even further.

### BTEX biodegradation in a liquid medium system

**Figure 1:**
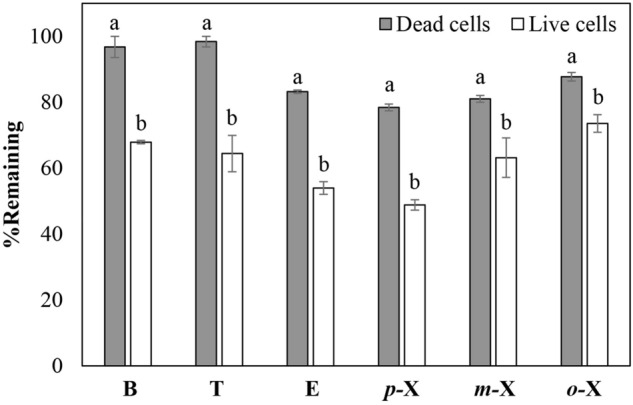
Percent remaining (%C/C_0_) of each BTEX compound (10 mg/L) when supplemented individually in the liquid medium system (MM, pH 7) containing dead cells and live cells of *B. amyloliquefaciens* subsp. *plantarum* W1 after 24 h in a shaking condition (200 rpm) at 30 °C. Different alphabet above the bars of the same compound indicates a significant difference at α = 0.1.

The ability of strain W1 to utilize each individual BTEX component as a sole carbon source was investigated in detail. As BTEX loss can be contributed not only by microbial activity, but also from evaporation, abiotic reactions and absorption onto the cells (both live and dead cells), the ‘dead cell control’ has been conducted in parallel in order to distinguish the loss by microbial activity from those of ‘abiotic losses’. The percentage of remaining BTEX (%C/C_0_) between live cells and dead cells were compared (Fig. 1). After 24 h, the remaining BTEX compounds in the live cells system were significantly lower than those of the dead cells control, indicating that *B. amyloliquefaciens* W1 was capable of degrading all BTEX compounds as a single substrate.

It should be noted that the ability to degrade all six BTEX compounds individually was not common even in the BTEX-degraders reported previously. *Pseudomonas putida* F1, a well-known aromatic degrader, was able to degrade only benzene, toluene and ethylbenzene^28^. Similarly, *Pseudomonas* sp. OX-1 can utilize only benzene, toluene and *o-*xylene as a sole carbon source^17^. Only a few bacterial strains were reported for their ability to degrade all BTEX compounds individually, for example, *Rhodococcus rhodochrous*^29^*, Pseudoxanthomonas spadix* BD-a59^30^, *Comamonas* sp. JB^31^ and *Microbacterium esteraromaticum* SBS1-7^20^. The percentage of BTEX biodegradation by strain W1 (at 24 h) were 29 ± 2.7, 34 ± 7.1, 29 ± 1.4, 30 ± 0.6, 18 ± 5.0 and 14 ± 4.0% for benzene, toluene, ethylbenzene, *p-*xylene, *m-*xylene and *o-*xylene, respectively (Table S2, supplementary material online). Therefore, the substrate preference of *B. amyloliquefaciens* W1 can be ranked as follows: toluene > benzene, ethylbenzene, *p-*xylene > *m-*xylene > *o-*xylene.

Ethylbenzene and/or *o-*xylene were frequently reported to be the most recalcitrant compounds in BTEX biodegradation^15,32,33^. Their presence frequently resulted in lower growth^15^ and/or BTEX degradation rate^29,30^. Although the toxicity exhibited by *o-*xylene was not observed in this study, it should be noted that the concentration tested (10 mg/L) was lower than the inhibition concentration mentioned in the relevant literature (e.g. 100 mg/L reported for *Ralsotonia* sp. PHS1^34^). Interestingly, strain W1 could effectively utilize ethylbenzene and approximately 29% of ethylbenzene could be degraded within 24 h (Fig. 1).

**Figure 2:**
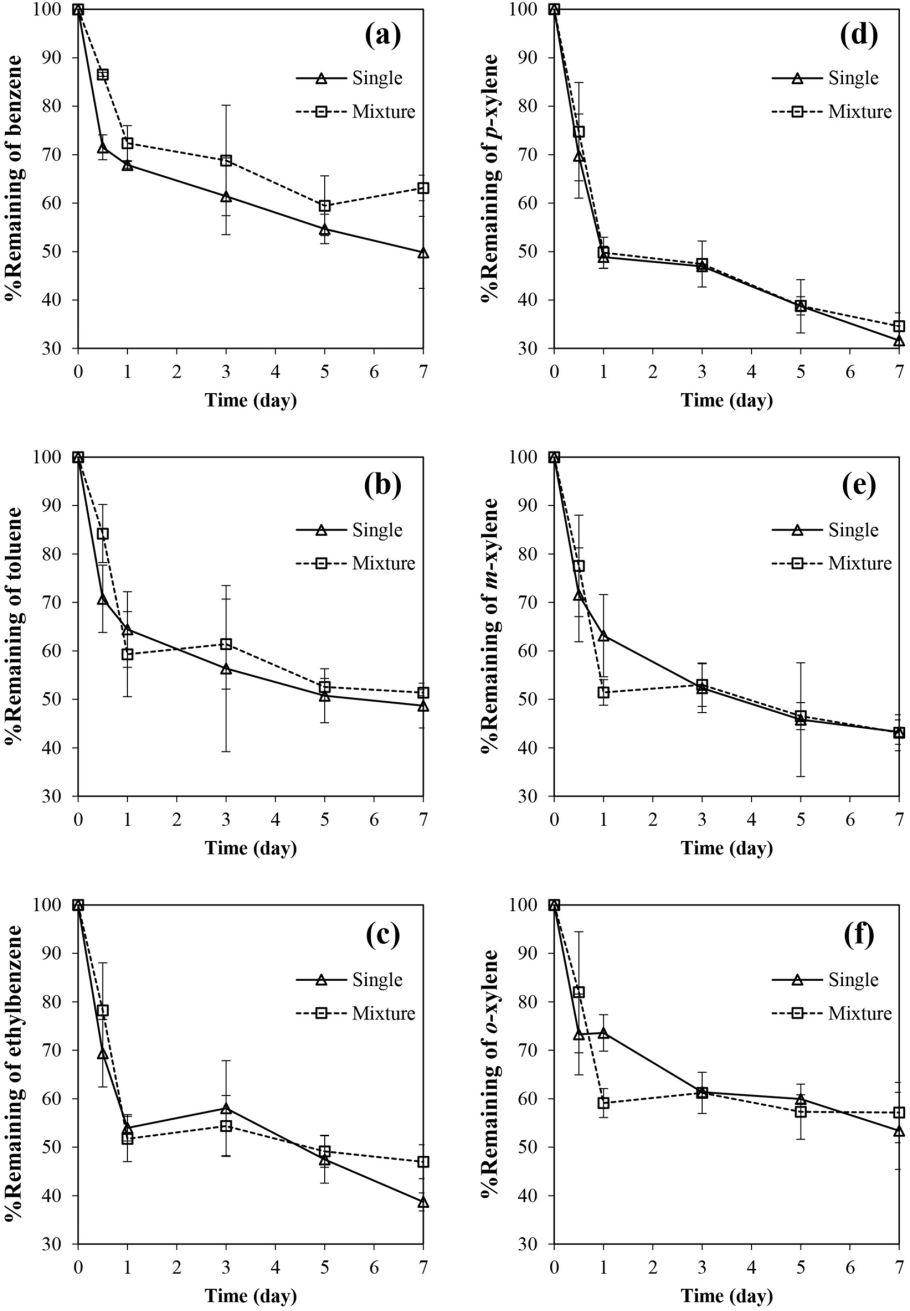
Biodegradation of each BTEX compound by *B. amyloliquefaciens* subsp. *plantarum* W1 in a liquid medium system when the compound was supplemented as an individual compound and a BTEX mixture; (**a**) benzene, (**b**) toluene, (**c**) ethylbenzene, (**d**) *p-*xylene, (**e**) *m-*xylene and (**f**) *o-*xylene. The biodegradation test was conducted using MM (pH 7) in a shaking condition (200 rpm) at 30 °C. Error bar indicates a standard deviation from 3 independent experiments.

In order to investigate the synergistic/inhibitory effect in the presence of other BTEX compounds, the biodegradation period in the liquid medium system was extended over a period of 1 week (Fig. 2). When BTEX was presented as a single substrate, all compounds could be rapidly degraded by approximately 30% within the first 12 h of incubation. During this initial period of 12 h, biodegradation of all BTEX components decreased by 5–15% when BTEX were supplemented as a mixture (Fig. 2; Table S3, supplementary material online). This result is consistent with the literature reporting the retardation of degradation rate in the presence of other BTEX compounds^34^.

It should be noted that the biodegradation of most BTEX compounds (except benzene) in a BTEX mixture became equal or even greater than those of a single substrate scenario once the adaptation period was over (Fig. 2; Table S3, supplementary material online). Biodegradation of *m-*xylene and *o-*xylene were significantly enhanced in a system supplemented with a BTEX mixture (Fig. 2e,f), indicating by 12–15% improvement in the percentage of biodegradation after 24 h of incubation (Table S3, supplementary material online). This result is consistent with the previous observation that the presence of benzene, toluene and ethylbenzene (BTE) enhanced xylene degradation in *Pseudomonas putida* YNS1^14^. This is not surprising considering that toluene, *m-*xylene and *o-*xylene degradation generated a common metabolite in the form of 3-methylcatechol (3-MC). 3-MC was broken down further into *cis*,*cis*-2-hydroxyl-6-oxohepta-2,4-dienoate and later on into other metabolites entering into the TCA cycle. Therefore, the presence of toluene or other metabolites that can enhance the degradation of 3-MC would also benefit the degradation of *m-*xylene and *o-*xylene. One possible explanation for this may be an induction effect of toluene or its corresponding metabolites toward an oxygenase-related enzyme in strain W1. Toluene and benzoate (a product of toluene degradation) were, for example, reported as the inducers for xylene monooxygenase (XMO) which played important roles in toluene and *m-*xylene biodegradation^35^. The addition of *o-*xylene, on the other hand, was reported to upregulate an expression of naphthalene 1,2-dioxygeanse (NDO; *nidABEF*) which was responsible for *o-*xylene, *m-*xylene, *p-*xylene as well as ethylbenzene utilization in *Rhodococcus opacus* TKN14^36^.

The inhibition of other BTEX compounds toward benzene biodegradation by strain W1 was also observed in this study (Fig. 2a; Table S3, supplementary material online). A similar phenomenon in which an inhibition of benzene degradation (approximately 70% reduction in benzene degradation rate) was observed in the presence of other BTEX compounds, especially ethylbenzene, was reported for *Stenotrophomonas maltophilia* T3-c^37^. The presence of ethylbenzene (at 20 mg/L) also resulted in a lower BTX biodegradation rate in a toluene-enriched microbial consortium containing *Rhodocoocus rhodochrous*^29^. Moreover, benzene and toluene degradation were also inhibited when *p-*xylene (50 mg/L) was added into the liquid medium system containing a pure culture of *Pseudomonas* sp. CFS-215^38^. Similarly, toluene degradation by a toluene-enriched consortium was retarded by 5–10 h when toluene was supplemented as a mixture with benzene, ethylbenzene and *p-*xylene^39^. A significant decrease in BTEX biodegradation rate was observed in *P. spadix* BD-a59 when ethylbenzene and *o-*xylene were presented in the liquid medium^30^. Interestingly, biodegradation of toluene, ethylbenzene and *p-*xylene by strain W1 seemed to be unaffected by the presence of other BTEX compounds (Fig. 2b–d). Such effective ethylbenzene degradation in strain W1 is suspected to reduce the strong inhibition effect of ethylbenzene observed by several other researchers mentioned previously.

The first-order kinetic model represented the BTEX biodegradation in the liquid medium system in terms of specific degradation constant (*k*) and half-life (*t*_*1/2*_) (Table S4, supplementary material online). The values of first-order degradation rate constant were 0.016 h^−1^ for benzene, 0.019 h^−1^ for toluene, 0.026 h^−1^ for ethylbenzene, 0.030 h^−1^ for *p-*xylene, 0.019 h^−1^ for *m-*xylene and 0.014 h^−1^ for *o-*xylene, corresponding to a half-life ranging from 23 to 48 h. The obtained *k* values were comparable with those reported in the literature, for example, *k* value of 0.0053, 0.0055 and 0.0027 h^−1^ were reported for benzene biodegradation by *Achromobacter* sp. AIEB-7, *Pseudomonas* sp. AIEB-4 and *Alcaligenes* sp. AIEB-6, respectively^40^. In summary, *B. amyloloquefaciens* subsp. *plantarum* W1 could effectively degrade all BTEX compounds supplemented individually or as a mixture. Therefore, the strain would be a good candidate for a bioremediation of BTEX-contaminated industrial exhaust stream and/or BTEX-contaminated wastewater which usually consists of several BTEX compounds in different combination.

### BTEX biodegradation in a soil slurry system

BTEX biodegradation activity in a liquid medium system and a soil slurry system were compared in a 30-day experiment supplemented with 60 mg/L of BTEX mixture. Generally, the reported concentration of BTEX are in the range of 0.1 to 100 µg/L (ppb) for typical groundwater. The BTEX concentration, however, could be as high as 3,500 µg/L for a contaminated groundwater^41^. Therefore, it should be noted that the range of BTEX concentrations employed in this study is significantly greater than actual BTEX concentrations found in the environment.

**Figure 3:**
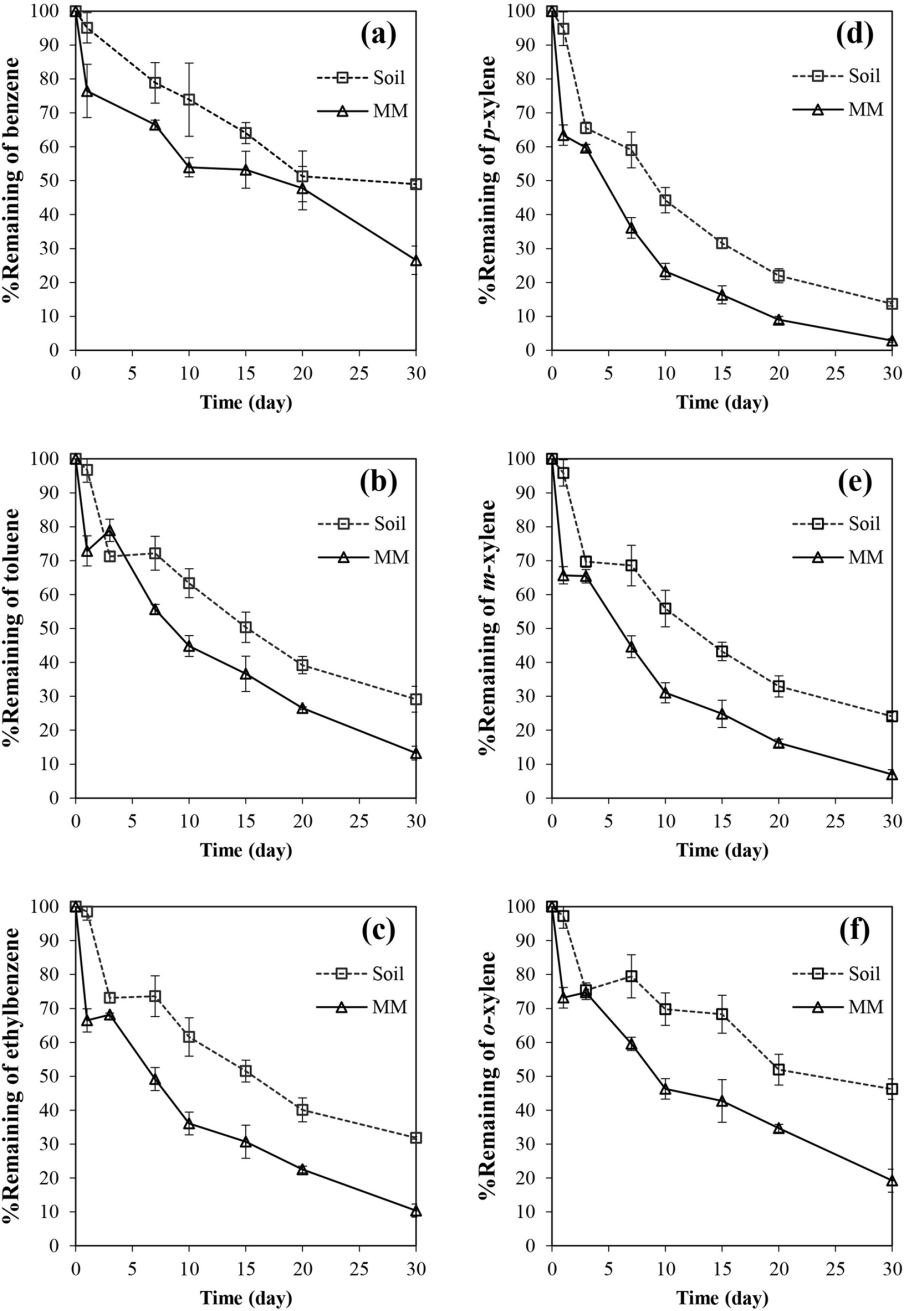
Biodegradation of each BTEX compound by *B. amyloliquefaciens* subsp. *plantarum* W1 in a liquid medium and a soil slurry system when BTEX was supplemented as a mixture; (**a**) benzene, (**b**) toluene, (**c**) ethylbenzene, (**d**) *p-*xylene, (**e**) *m-*xylene and (**f**) *o-*xylene. The biodegradation test was conducted using MM (pH 7) in a shaking condition (200 rpm) at 30 °C. Error bar indicates a standard deviation from 3 independent experiments.

By comparing these two systems, the liquid medium system exhibited greater level of overall BTEX degradation (Fig. 3). Catabolic repression from soil nutrients was suspected as a ‘probable’ cause, as the soil organic matters with hydrophobic characteristics could absorb BTEX compounds into the soil particles^42^. Evidence of catabolic repression by an alternative carbon source was also reported in benzene biodegradation by *Ralstonia pickettii* PKO1^43^. Nonetheless, it should be noted that the effects of soils on BTEX biodegradation is still inconclusive. While a decrease in BTEX biodegradation rate after an addition of soil has been reported^44^, an addition of organic compounds derived from soil provided a nutritional benefit for growth of *P. spadix* BD-a59 and thus contributed to the higher BTEX biodegradation rate observed^30^. It should be noted that the dependency of *P. spadix* BD-a59 on the organic nutrients (unidentified nutrients presented in the solid portion of soil as well as in a yeast extract) may contribute to the difference observed.

In this study, the viable cell count (CFU/mL) and spore count (spores/mL) in both systems were monitored during an entire experimental period. While a total count of approximately 10^8^ CFU/mL could be maintained in both systems, a percentage of sporulation (percentage of cells with spore formation) increased drastically and reached 100% within 3 days. This observation agreed with the fact that BTEX degradation rate was fastest during the first 3 days (Fig. 3). Despite a complete sporulation, a continuous decrease in residual BTEX could be observed up to 30 days. Sporulation might be resulted from nutrient depletion in MM considering the fact that MM was a minimal medium containing only inorganic salts. Such nutrient limitation was reported to trigger sporulation in genus *Bacillus*^45,46^.

In the liquid medium system, most of the BTEX compounds except benzene and *o-*xylene could be effectively removed to less than 10% within 30 days. This result agreed with the lower preference toward *o-*xylene (Fig. 1) as well as the previously described inhibitory effect of other BTEX compounds on benzene biodegradation (Fig. 2). With a significantly lower degradation observed, the residual BTEX in the soil slurry system was between 13.7% (in case of *p-*xylene, Fig. 3d) and 49.0% (in case of benzene, Fig. 3a). Therefore, the presence of soil matrix resulted in a significantly slower BTEX degradation in *B. amyloliquefaciens* W1 (Table S4 and S5, supplementary material online).

### Proposed BTEX biodegradation pathways in B. amyloliquefaciens W1

During an aerobic BTEX biodegradation, aromatic compounds were incorporated with an oxygen atom via activity of mono- or dioxygenases^47^. Analyses of the metabolites produced during the biodegradation by solvent extraction and identification of the metabolites by LC–MS/MS allowed us to postulate the BTEX biodegradation pathways utilized by the target strain-of-interest. The pathway construction was performed based on the previously reported enzymatic reactions from the University of Minnesota Biocatalysis/Biodegradation Database (UMBBD, https://umbbd.msi.umn.edu)^48^ as well as from other relevant literature.

**Figure 4:**
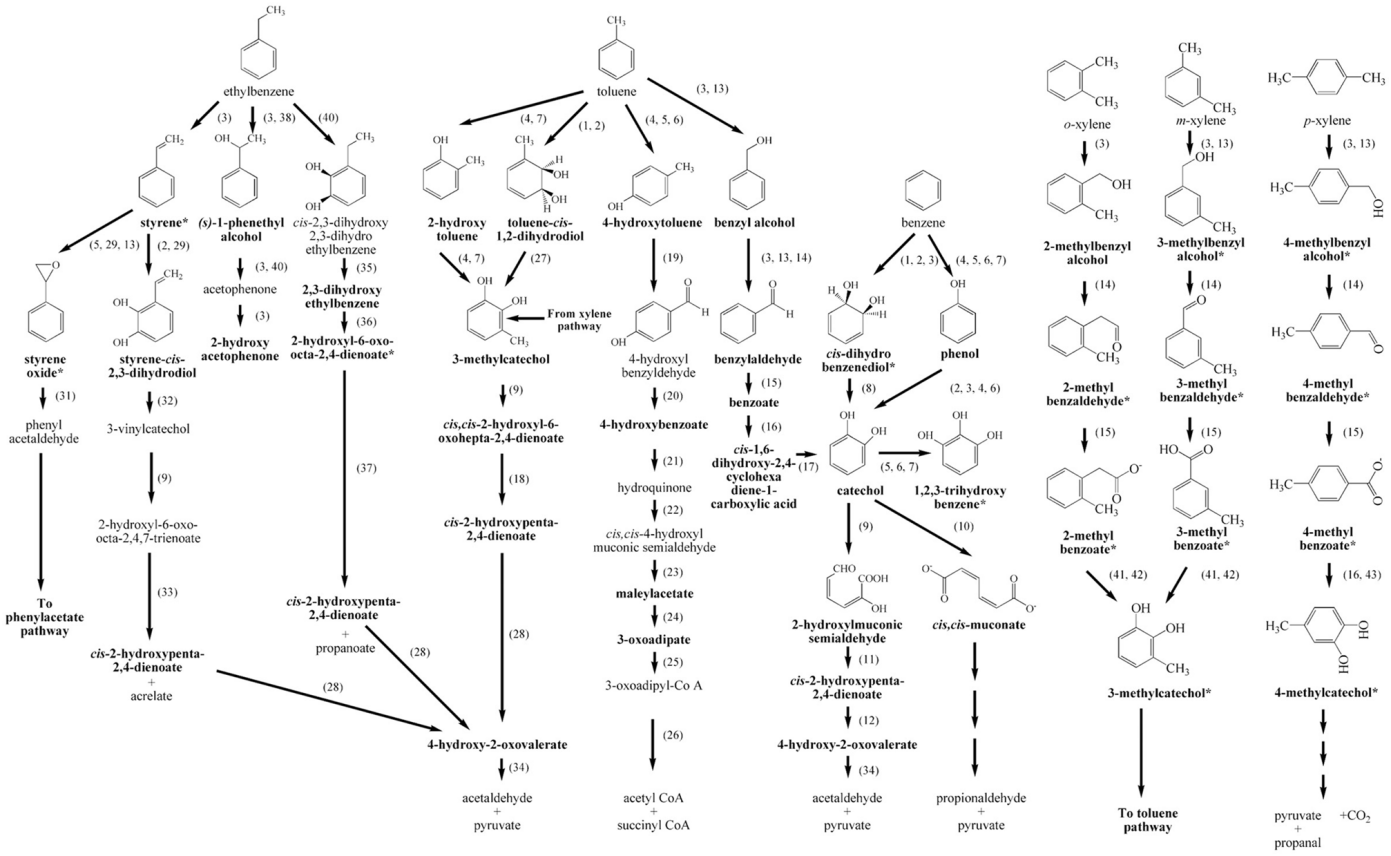
Proposed BTEX biodegradation pathways in *B. amyloliquefaciens* subsp. *plantarum* W1. Putative enzymes responsible for the reaction are shown in the bracket. The detected metabolite (*bold alphabet*) with high signal intensity is indicated by an asterisk. The putative enzymes responsible for BTEX degradation are presented as follow; (1) benzene 1,2-dioxygenase or BDO [E.C.1.14.12.3], (2) toluene dioxygenase or TDO [E.C.1.14.12.11], (3) naphthalene 1,2-dioxygenase or NDO [E.C.1.14.12.12], (4) phenol hydroxylase or PH [E.C.1.14.13.7], (5) toluene 4-monooxygenase or T4MO [E.C.1.14.13.236], (6) toluene 3-monooxygenase or T3MO, (7) toluene *ortho-*monooxygenase or TOM [E.C.1.14.13.243], (8) *cis*-1,2-dihydrobenzene-1,2-diol dehydrogenase [E.C.1.3.1.19], (9) catechol 2,3-dioxygenase or C23DO [E.C.1.13.11.2], (10) catechol 1,2-dioxygenase or C12DO [E.C.1.13.11.1], (11) 2-hydroxymuconate-6-semialdehyde hydrolase [E.C.3.7.1.9], (12) 2-oxopent-4-enoate hydratase [E.C. 4.2.1.80], (13) xylene monooxygenase or XMO [E.C.1.14.15.-], (14) aryl-alcohol dehydrogenase [E.C.1.1.1.90], (15) benzaldehyde dehydrogenase [E.C.1.2.1.28], (16) benzoate 1,2-dioxygenase [E.C.1.14.12.10], (17) 1,6-dihydroxycyclohexa-2,4-diene-1-carboxylate dehydrogenase [E.C.1.3.1.25], (18) 2-hydroxy-6-oxohepta-2,4-dienoate hydrolase [E.C.3.7.1.25], (19) 4-hydroxybenzaldehyde dehydrogenase [E.C.1.2.1.64], (20) 4-hydroxybenzaldehyde dehydrogenase [E.C.1.2.1.64], (21) 4-hydroxybenzoate 1-hydroxylase [E.C.1.14.13.64], (22) hydroquinone dioxygenase [E.C.1.13.11.-], (23) 4-hydroxymuconic semialdehyde dehydrogenase [E.C.1.2.1.61], (24) maleylacetate reductase [E.C.1.3.1.32], (25) 3-oxoadipate CoA-transferase [E.C.2.8.3.6], (26) acetyl-CoA C-acyltransferase [E.C.2.3.1.16], (27) toluene dihydrodiol dehydrogenase [E.C.1.3.1.19], (28) 2-oxopent-4-enoate hydratase [E.C.4.2.1.80], (29) styrene monooxygenase or SMO [E.C.1.14.14.11], (30) styrene dioxygenase or SDO [E.C.1.14.12.-], (31) styrene oxide isomerase [E.C.5.3.99.7], (32) *cis*-benzene glycol dehydrogenase [E.C.1.3.1.19], (33) 2-hydroxymuconate-6-semialdehyde hydrolase [E.C.3.7.1.9], (34) 4-hydroxy-2-oxovalerate aldolase [E.C.4.1.3.39], (35) *cis*-dihydroethylcatechol dehydrogenase [E.C.1.3.1.66], (36) 2,3-dihydroxy-ethylbenzene 1,2-dioxygenase [E.C.1.13.11.-], (37) 2-hydroxy-6-oxo-octa-2,4-dienoate hydrolase [E.C.3.7.1.-], (38) ethylbenzene hydroxylase or EBH [E.C.1.17.99.2], (39) (*S*)-1-phenylethanol dehydrogenase [E.C.1.1.1.311], (40) ethylbenzene dioxygenase or EBDO [E.C.1.14.12.-], (41) toluate dioxygenase [E.C.1.14.12.-], (42) 1,2-dihydroxy-6-methylcyclohexa-3,5-dienecarboxylate dehydrogenase [E.C.1.3.1.68], and (43) 4-methylcyclohexa-3,5-diene-1,2-*cis*-diol-1-carboxylic acid dehydrogenase [E.C.1.3.1.67].

The ability to utilize BTEX in genera *Bacillus* is not fully elucidated as a result of its minority status in BTEX-enriched culture^49^. Only a few *Bacillus* strains were reported as BTEX degraders, for example, *B. subtilis* DM-04 (able to degrade benzene, toluene and *m-*xylene)^50^, *B. stratosphericus* FLU-5 (capable of growing on toluene, ethylbenzene, *o-*xylene, *m-*xylene and *p-*xylene)^51^ and *B. cereus* ATHH39 (able to degrade toluene)^52^. Thus far, only *Bacillus pumilus* MVSV3 has been reported for its mono-oxidation (for toluene and ethylbenzene) and di-oxidation (for benzene and *o-*xylene)^22^. In this study, biodegradation pathways of all six BTEX compounds in *B. amyloliquefaciens* were proposed for the first time (Fig. 4).

When benzene was supplemented as a sole carbon source for *B. amyloliquefaciens* W1, the following intermediates were detected: benzene dihydrodiol, phenol, catechol, 2-hydroxymuconic semialdehyde, *cis,cis-*muconate, 1,2,3-trihydroxybenzene, *cis-*2-hydroxypenta-2,4-dienoate and 4-hydroxy-2-oxovalerate (Table S6, supplementary material online). Two main existing pathways were observed as benzene was converted to (1) benzene dihydrodiol via di-oxidation and (2) phenol via mono-oxidation. Several enzymes were reported for dihydroxylation of benzene including benzene 1,2-dioxygenase (BDO; E.C.1.14.12.3) from *Pseudomonas putida* ML2^53^, toluene dioxygenase (TDO; E.C.1.14.12.11) from *P. putida* F1^54^ and naphthalene dioxygenase (NDO; E.C.1.14.12.12) from *Pseudomonas* sp. strain NCIB 9816-4^55^.

Phenol was reported as a metabolite in benzene biodegradation by an activity of several enzymes including toluene 4-monooxygenase (T4MO; E.C.1.14.13.236) from *Pseudomonas mendocina* KR1^56^, toluene 3-monooxygenase (T3MO) from *Ralstonia pickettii* PKO1^57^, phenol hydroxylase (PH; E.C.1.14.13.7) and toluene *ortho-*monooxygenase (TOM; E.C.1.14.13.243) from *Burkholderia cepacia* G4^58^. Then, catechol or substituted catechol was further metabolized by either catechol 1,2-dioxygenase (C12DO; E.C.1.13.11.1) or catechol 2,3-dioxygenase (C23DO; E.C.1.13.11.2) and further converted to pyruvate or acetaldehyde which entered into the TCA cycle^59^. Although activities of both mono-oxidation and di-oxidation were detected in *B. amyloliquefaciens* W1, the relatively higher signal of benzene-*cis-*dihydrodiol was observed, suggesting that the biodegrading activity via di-oxidation was probably more active in strain W1. This is not surprising considering that di-oxidation is more frequently reported in aerobic bacteria than mono-oxidation^60–62^.

Four different upper pathways out of five previously reported toluene biodegradation pathways were observed in *B. amyloliquefaciens* W1, considering the fact that the following metabolites were detected: benzyl alcohol, 2-hydroxytoluene, 4-hydroxytoluene, toluene-*cis*-dihydrodiol , benzaldehyde, benzoate, *cis-*1,6-dihydroxy-2,4-cyclohexadiene-1-carboxylic acid, catechol, 3-methylcatechol, *cis,cis*-2-hydroxy-6-oxohepta-2,4-dienoate, *cis*-2-hydroxypenta-2,4-dienoate, 4-hydroxybenzoate, maleylacetate and 3-oxoadipate (Table S7, supplementary material online). Four different oxygenated toluenes were detected, including (1) mono-oxidation at a methyl group of toluene to produce benzyl alcohol; (2) mono-oxidation at an *ortho-* position to produce 2-hydroxytoluene; (3) mono-oxidation at a *para-* position of toluene to produce 4-hydroxytoluene and (4) di-oxidation to produce a corresponding *cis*-dihydrodiol (toluene-*cis-*dihydrodiol).

Benzyl alcohol was reported as a product from the toluene oxidation by xylene monooxygenase (XMO; E.C.1.14.15.-) in TOL plasmid pWW0 of *P. putida* mt-2^63^ and naphthalene 1,2-dioxygenase (NDO) in *Pseudomonas* sp. strain NCIB 9816–4^64^. It was further oxidized to benzaldehyde, benzoate and finally entered into the benzene pathway via catechol. 2-Hydroxytoluene or *o-*cresol was further oxidized to 3-methylcatechol, which could be metabolized through the central metabolic pathway via 4-hydroxy-2-oxovalerate. Several enzymes were reported to convert toluene into 2-hydroxytoluene including phenol hydroxylase (PH) of *Arthrobacter* sp. W1^65^ and toluene-*ortho*-monooxygenase (TOM) of *B. cepacia* G4^66^. Another toluene biodegradation route observed in *B. amyloliquefaciens* W1 was the mono-oxidation through 4-hydroxytoluene or *p-*cresol. This conversion was observed as an activity of toluene 4-monooxygenase (T4MO) in *P. mendocina* KR1^67^. Besides T4MO, T3MO from *R. pickettii* PKO1^68^ and PH from *Arthrobacter* sp. W1^65^ were also reported for toluene conversion through *p-*cresol pathway. Another proposed toluene biodegradation pathway in strain W1 was the di-oxidation via toluene-*cis-*dihydrodiol which was reported as an activity of toluene 1,2-dioxygenase (TDO; E.C.1.14.12.11)^53,69^ and/or benzene 1,2-dioxygenase (BDO; E.C. 1.14.12.3) from *P. putida* F1^70^.

Three ethylbenzene biodegradation pathways were found in *B. amyloliquefaciens* W1 when ethylbenzene was supplemented as a sole carbon source. Several metabolites were detected including styrene, *(s)-*1-phenethyl alcohol, styrene oxide, styrene-*cis-*dihydrodiol, *cis-*2-hydroxypenta-2,4-dienoate, 2-hydroxy ethylbenzene, 2-hydroxy-6-oxo-octa-2,4-dienoate and *cis*-2-hydroxypenta-2,4-dienoate (Table S8, supplementary material online). Based on the detected metabolites, naphthalene 1,2-dioxygenase (NDO) or other enzymes with a similar activity seemed to play an important role in ethylbenzene biodegradation by strain W1. According to a study on the activities of a purified NDO toward ethylbenzene^64^, 1-phenethyl alcohol and styrene were formed via a monohydroxylation and a desaturation at ethyl group of ethylbenzene, respectively. (*S*)-1-Phenethyl alcohol was further oxidized to acetophenone and 2-hydroxyacetophenone while styrene was further converted to styrene oxide and styrene*-cis-*dihydrodiol. Styrene monooxygenase (SMO; E.C.1.14.14.11) from *P. putida* S12^71^, xylene monooxygenase (XMO; E.C.1.14.15.-) from TOL plasmid pWW0 in *P. putida* mt-2^72^ and T4MO from *P. mendocina* KR1^73^ were reported for their ability to carry out an epoxidation of styrene to styrene oxide. Styrene-*cis-*dihydrodiol, on the other hand, has been reported as a product of styrene dioxygenase (SDO; E.C.1.14.12.-) from *Rhodococcus rhodochrous* NCIB 13259^74^ and toluene dioxygenase (TDO) from *P. putida* F39/D^75^. As 2,3-dihydroxyethylbenzene was detected in this study, another putative ethylbenzene biodegradation pathway via an activity of ethylbenzene dioxygenase (EBDO; E.C.1.14.12.-), as observed in *Rhodococcus jostii* RHA1^76^, was included in the proposed pathways (Fig. 4). Interestingly, a relatively higher concentration of metabolites was observed in the styrene pathway in strain W1. This observation suggested highly effective desaturation and epoxidation which could play an important role in ethylbenzene biodegradation through styrene oxide (later entered into the phenylacetate pathway).

*B. amyloliquefaciens* W1 could utilize all three isomers of xylene and several metabolites were detected from each xylene biodegradation pathway including 2-methylbenzyl alcohol, 2-methylbenzaldehyde, 2-methylbenzoate and 3-methylcatechol from *o-*xylene (Table S9, supplementary material online), 3-methylbenzyl alcohol, 3-methylbenzoate, and 3-methylcatechol from *m*-xylene (Table S10, supplementary material online), and 4-methylbenzyl alcohol, 4-methylbenzaldehyde, 4-methylbenzoate, and 4-methylcatechol from *p*-xylene (Table S11, supplementary material online). Based on the detected metabolites, all isomers of xylene shared a similar pathway in which xylenes were oxidized to the corresponding alcohols, aldehydes and benzoates. 2-Methylbenzoate (from an oxidation of *o-*xylene) and 3-methylbenzoate (from an oxidation of *m-*xylene) were further oxidized to 3-MC which was metabolized via toluene pathway as previously described. 4-Methylbenzoate, from an oxidation of *p-*xylene, can be further metabolized to 4-methylcatechol, which was cleaved by catechol 2,3-dioxygenase (C23DO) or catechol 1,2-dioxygenase (C12DO) and then further converted into metabolites that could enter into the TCA cycle.

Purified naphthalene 1,2-dioxygenase (NDO) from *Pseudomonas* sp. strain NCIB 9816-4 was reported to convert all three xylene isomers into the substituted benzyl alcohols and benzaldehyde derivatives^64^. Xylene monooxygenase (XMO) from *P. putida* mt-2, on the other hand, could catalyze only *p-*xylene and *m-*xylene^77^. The fact that the conversion of xylene into the substituted benzyl alcohol was observed in all xylene isomers suggested that the xylene degradation in strain W1 was most likely a result of NDO-like enzyme. Previously, evidence of NDO-like gene was reported in *Bacillus megaterium* strain 2 and strain 3 based on a PCR and hybridization analysis using *ndo*B gene. Naphthalene, however, was not degraded by those *B. megaterium* strains, indicating a knowledge gap for NDO-like enzyme in genus *Bacillus*^78^. Interestingly, a significantly higher signal of metabolites, including corresponding benzaldehydes and corresponding benzoates, was detected in *m-*xylene and *p-*xylene degradation by *B. amyloliquefaciens* W1. This observation suggested a high metabolic flux in the pathways which agreed well with the high degradation observed in *m-*xylene and *p*-xylene (Figs. 1, 2, 3). The fact that *o-*xylene was the least favorable substrate in strain W1 suggested the presence of a bottleneck in the upper part of *o-*xylene pathway (prior to 3-MC).

## Conclusion

*B. amyloliquefaciens* W1, isolated from a petrochemical waste, was able to degrade all six BTEX compounds presented as an individual compound or a BTEX mixture. Based on the presence of *amy*E-like gene, strain W1 belongs to the subsp. *plantarum* which has been frequently reported in association with plants. Although the presence of soil matrix resulted in a significantly slower BTEX degradation, *B. amyloliquefaciens* strain W1 could effectively remove BTEX in both liquid medium and soil slurry system. To the best of our knowledge, this study was the first direct evidence of a complicated BTEX-biodegrading pathway in the genera *Bacillus.* Considering its safe status and the ability to thrive in most soil ecosystems, *B. amyloliquefaciens* W1 would be considered beneficial for in situ bioremediation applications. The well-documented ability of *B. amyloliquefaciens* subsp. *plantarum* to grow in association with plants also suggests that strain W1 should be evaluated for its biodegrading ability in the rhizosphere system. Additionally, strain W1 only requires simple cultivation conditions, and therefore can be easily produced on large-scale for scale-up study or commercialization.

## Methods

### Chemicals and media

Chemicals used in this study, including benzene (99.8% purity), toluene (99.5% purity), ethylbenzene (> 98% purity), *p-*xylene (> 98% purity), *o-*xylene (> 98% purity) and *m-*xylene (> 98% purity), were of GC grade (developed specifically for residue analysis of pesticides with very low content of non-volatile matters) from well-known manufacturers. Mineral salts medium (MM)^20^ was used for an enrichment and biodegradation test. It consisted of (per L) 0.91 g of KH_2_PO_4_, 0.4 g of K_2_HPO_4_, 2.39 g of Na_2_HPO_4_·2H_2_O, 2.96 g of KNO_3_, 1.97 g of (NH_4_)_2_SO_4_, 2 g of MgSO_4_·7H_2_O, 0.2 g of FeSO_4_·7H_2_O, 0.5 g of NaHCO_3_, 0.54 g of MnSO_4_·H_2_O, 0.04 g of ZnSO_4_·7H_2_O, 2.26 g of CaCl_2_, 1 g of Na_2_MoO_4_·2H_2_O, 0.04 g of CoCl_2_·6H_2_O and 8.86 g of Na_2_EDTA·2H_2_O. The pH of MM was adjusted to 6.8–7.0 with NaOH and HCl before use.

### Isolation of BTEX-degrading bacteria

BTEX-degrading bacteria were isolated using an enrichment protocol. The enrichment experiment was conducted in a 25 mL-serum bottle sealed with a butyl rubber septum and an aluminum cap. Briefly, MM supplemented with 1,200 mg/L of a BTEX mixture (200 mg/L of each compound) was inoculated with 1% (v/v) of a liquid petrochemical waste and incubated at 30 °C, 120 rpm. Bacterial culture was transferred into a fresh MM supplemented with the same level of BTEX mixture every 3 days for 1 month. After that, the culture was plated onto a tryptic soy agar (TSA) plate. All bacterial colonies with different morphologies (e.g. form, elevation, margin, color and transparency) were collected and streaked on a fresh TSA plate repeatedly until the pure isolates (exhibiting only one colony morphology) were obtained. Glycerol stocks were prepared and kept at − 80 °C for a long-term storage.

### Strain identification and characterization

16S rDNA sequence was used to identify the obtained bacterial isolate^79^. Genomic DNA was extracted using HiYield Genomic DNA Mini Kit (RBC Bioscience, Taiwan) according to the manufacturer's recommendation. 16S rDNA fragment was amplified by PCR using the universal primers 27F (5-AGAGTTTGATCMTGGCTCAG-3′) and 1492R (5′-TACGGYTACCTTGTTACGACTT-3′). The temperature profile used were as follows: 25 cycles of denaturation at 95 °C for 1 min, annealing at 55 °C for 1 min, and extension at 72 °C for 1.5 min followed by 1 cycle of a final extension at 72 °C for 5 min. The 16S rDNA fragment obtained was purified using a HiYield Gel/PCR DNA Fragments Extraction Kit (RBC Bioscience, Taiwan) and sequenced by Macrogen (Seoul, Korea). The sequence was then compared with the data in GenBank using the basic local alignment search tool (BLAST) of the National Center for Biotechnology Information (NCBI). As *Bacillus amyloliquefaciens* subsp. *amyloliquefaciens* and *Bacillus amyloliquefaciens* subsp. *plantarum* can be distinguished by the presence of a specific polysaccharide-degrading gene, 2 different sets of primers designed specifically for a starch-liquefying α-amylase (*amy*A) and a saccharifying enzyme (*amy*E) (Table S1, supplementary material online) were used for subspecies identification. The temperature profile used were as follows: 25 cycles of denaturation at 95 °C for 1 min, annealing at 56 °C for 1 min, and extension at 72 °C for 2 min followed by 1 cycle of a final extension at 72 °C for 5 min. PCR products were visualized on 1% agarose gel with a post-staining using Fluorovue Nucleic Acid Gel Stain (SMOBIO, Taiwan).

### Quantification of BTEX-biodegrading activity in a liquid medium system

BTEX-biodegrading activity in a liquid medium system was performed according to the protocol described by Wongbunmak et al.^20^. Briefly, *B. amyloliquefaciens* W1 was cultivated in a lysogenic broth (LB) at 30 °C, 200 rpm for 15 h, then the cells were harvested by centrifugation (Allegra X-30R, Beckman, USA) and washed twice with a sterile normal saline solution (NSS). The harvested cells were re-suspended in MM to obtain a cell suspension with an OD_600_ of 5 (corresponding to 1.6 × 10^8^ CFU/mL). BTEX, diluted with dimethyl sulfoxide (DMSO), was supplemented into 1 mL of the cell suspension either as an individual component (at 10 mg/L) or a BTEX mixture (10 mg/L of each component for a total of 60 mg/L BTEX). The experiment was conducted in a glass vial with a PTFE/rubber septum and a screw cap in order to prevent the leakage of BTEX vapor. The BTEX-biodegrading activity of strain W1 was evaluated at 30 °C, 200 rpm for 7 days. A similar experimental set up with dead cells, treated with 2% (w/v) sodium azide (NaN_3_), was conducted in parallel as a negative control. Samples were sacrificed at a specified period for the detection of the remaining BTEX concentration by GC-FID (GC-2014, Shimadzu, Japan).

### Quantification of BTEX biodegrading activity in a soil slurry system

BTEX-biodegrading activity in a soil slurry system was performed according to the protocol described by Wongbunmak et al.^20^. An uncontaminated soil sample (pristine soil; silt, pH 6.5) was collected from an agricultural area in Ratchaburi Province, Thailand, in March of 2014. The soil particles were passed through an 18 × 18 mesh (opening = 1.429 mm) and then sterilized by autoclaving at 121 °C for 15 min (3 times) before use. The experimental set up consisted of 0.6 mL of the cell suspension mixed with 0.6 g of sterile pristine soil. The cell suspension used in this experiment was concentrated so that the soil slurry system contained an equal number of cells to that of the liquid medium system described above. Then, 60 mg/L of BTEX mixture was supplemented and the experiment was conducted in a similar manner to that of the liquid medium system.

### BTEX analysis by GC-FID

To quantify the remaining BTEX concentration in the system, *n-*hexane and 1-hexanol were used as an extracting solvent and an internal standard, respectively. The extracting solvent (0.2 mL) and the cell suspension (1 mL) were mixed vigorously for 5 min and then centrifuged at 11,000×*g*, 4 °C for 10 min. The solvent layer (1 µL) was injected into a gas chromatography (GC-2014, Shimadzu, Japan) equipped with a capillary DB-wax column (J&W Scientific, CA; 30 m-length, 0.250 mm-inner diameter, 0.25 µm-film thickness) and a flame ionization detector (FID) with split mode (10:1). The oven temperature was controlled as follows: held at 50 °C for 5 min; raise to 120 °C at a rate of 5 °C/min and then raise to 240 °C at a rate of 30 °C/min. Helium was used as a carrier gas at 17 mL/min of total flow rate. The injector and detector were operated at 250 °C and 300 °C, respectively. Known concentrations of standard BTEX were supplemented into the MM and extracted in a similar manner to that of the experiment set up. The obtained peak area was used to construct a standard curve between ‘Ratio of Area-BTEX and Area-internal standard’ and ‘BTEX concentration’. The standard curve of each BTEX compound (Fig. S2, supplementary material online) was constructed from different concentrations of standard solution (ranging from 0.5 to 100 mg/L). The same extraction and BTEX analysis protocol with that of the sample was carried out for standard solution. The coefficients of determination (R-square) were 0.997 for benzene, 0.999 for toluene, 0.998 for ethylbenzene, 0.998 for *p-*xylene, 0.997 for *m-*xylene and 0.996 for *o-*xylene. Remaining BTEX (%) in the system was calculated as %C/C_0_ when C was the actual BTEX concentration at a particular sampling time and C_0_ was the initial BTEX concentration. The results were presented as an average value from at least 3 independent experiments.

### First-order kinetics of BTEX biodegradation

The biodegradation of BTEX in a single substrate and a mixture condition could be described by the first-order kinetics^80^ using Eq. (1):

1 $$ C = C_{0} e^{ - kt} $$

where *C* is the BTEX concentration (mg/L) at a certain time, *C*_*0*_ is the initial concentration of BTEX (mg/L), *k* is the specific degradation rate constant (h^−1^) and *t* is the biodegradation period (h).

Biodegradation half-life time was calculated using Eq. (2):

2 $$ t_{1/2} = \frac{\ln 2}{k} $$

where *k* is the specific degradation rate constant calculated from Eq. (1).

### Viable cell and spore enumeration

At a specified sampling time, the cell suspension (200 µL) was taken from the system with a sterile syringe. The sample was serially diluted with sterile NSS. The viable cell count (CFU/mL) and spore count (spores/mL) were determined using a drop plate technique^81^. Briefly, 2 drops of the cell suspension (10 µL) from each dilution were dropped onto LB agar and allowed to dry. Plates were incubated at 30℃ and the number of colonies were counted after 16 h. For the spore count, the cell suspension was heated at 80℃ for 10 min to kill the vegetative cells prior to the enumeration.

### Data analysis

All experiments were conducted in triplicate. The data were presented as the mean of three replicates ± standard deviation of mean. ANOVA test and Tukey’s multiple comparison were performed using Minitab software (Release 15, State College, PA, USA) for the biodegradation data to compare that the sample means differ at a significant level *P* < 0.1.

### Metabolites tracking

The metabolites tracking protocol (Fig. S3, supplementary material online) was modified from that reported previously^20^. Briefly, the cell suspension was prepared as described above. Then, an individual BTEX component was supplemented into 20 mL of the cell suspension at 10% (v/v). The experiment was performed in a 100 mL serum bottle sealed with a butyl rubber septum and an aluminum cap. The degradation was performed at 30 °C for 3 h under shaking condition at 200 rpm. A sterile MM supplemented with individual BTEX component, referred as ‘Abiotic control’, was conducted in parallel. Metabolites were extracted by using 15 mL of ethyl acetate. After mixing by sonication for 10 min, the upper layer (ethyl acetate) was collected. The extraction was repeated twice and the solvent phase were pooled together. The residual water in the sample was removed by an addition of sodium sulfite. The sample was dried under vacuum (− 0.8 bar) for 1 h and then dried under nitrogen gas (flow rate of 15 L/min) for 5 min. The dried residue was resuspended in 200 µL of 5% (v/v) acetonitrile (ACN). The sample was filtered with 0.2 µm nylon filter before analysis.

Metabolites were analyzed by LC–MS/MS (Dionex UltiMate 3000/Bruker MicrOTOF/maXis) operated in a positive ionization mode (collision energy 20 eV; scan range 40–1500 m/z). Acclaim 120 C18 reversed phase column (2.2 µm, 2.1 × 100 mm) was used for separation. A binary gradient consisting of water (mobile phase A) and ACN (mobile phase B) (both + 0.1% formic acid) at a constant flow rate of 0.3 mL/min was used. A gradient elution was applied as follows: 0–1 min, 5% B; 1–9 min, 5–60% B; 9–12 min, 90% B and 12–15 min, 5% B. The signal from the negative control served as a ‘baseline’ for identifying the metabolites formed as a result of microbial activity. Each identified metabolite (peak) was then subjected to MS/MS analysis for chemical identification. The fragmentation pattern was compared with the MS/MS database of METLIN (The Scripps Research Institute). Biodegradation pathways as well as possible responsible enzymes in the biodegradation of each BTEX compound by *B. amyloliquefaciens* W1 were proposed according to the detected metabolites.

## Supplementary information

Supplementary Information.

## Data Availability

All data generated or analyzed during this study are included in this published article (and its Supplementary Information file online).
