# Supplementary material for: BTEX biodegradation by Bacillus amyloliquefaciens subsp. plantarum W1 and its proposed BTEX biodegradation pathways
Source: Sci Rep. 2020 Oct 15;10:17408. doi: 10.1038/s41598-020-74570-3 (PMC7562720; doi:10.1038/s41598-020-74570-3)
Supplement: Supplementary file 1 — Supplementary Information. [file 41598_2020_74570_MOESM1_ESM.pdf]

Supplementary Material Online

BTEX biodegradation by *Bacillus amyloliquefaciens* subsp. *plantarum* W1 and its proposed BTEX biodegradation pathways

Akanit Wongbunmak[a], Sansanee Khiawjan[a], Manop Suphantharika[a] and Thunyarat Pongtharangkul[a*]

[a]*Department of Biotechnology, Faculty of Science, Mahidol University, Bangkok, Thailand*

***Corresponding author:** Thunyarat Pongtharangkul,

Department of Biotechnology, Faculty of Science,
Mahidol University, Bangkok 10400, Thailand
Tel: +6686-379-7037

Email: thunyarat.pon@mahidol.ac.th

ORCID ID: 0000-0003-0019-4741

First author: Akanit Wongbunmak

Email: akanit.wong@gmail.com

Co-authors: Sansanee Khiawjan

Email: khsansanee@gmail.com

Manop Suphantharika
Email: manop.sup@mahidol.ac.th

Table S1 Specific primers for genes encoding polysaccharide-degrading enzymes

Primer Name	Size (bp)	Sequence (5' to 3')	Product (bp)
*amy*AF	35	GCC<u>TGTACA</u>ATGATTCAAAAACGAAAGCGGACAGT	1,545
*amy*AR	38	GCC<u>AGATCT</u>TTATTTCTGAACATAAATGGAGACGGACC	
*amy*EF	41	GCC<u>GGTACC</u>ATGTTTGAAAAACGATTCAAAACCTCTTTACT	1,980
*amy*ER	36	GCC<u>AGATCT</u>TTAATGCGGAAGATAACCATTCAAACC	

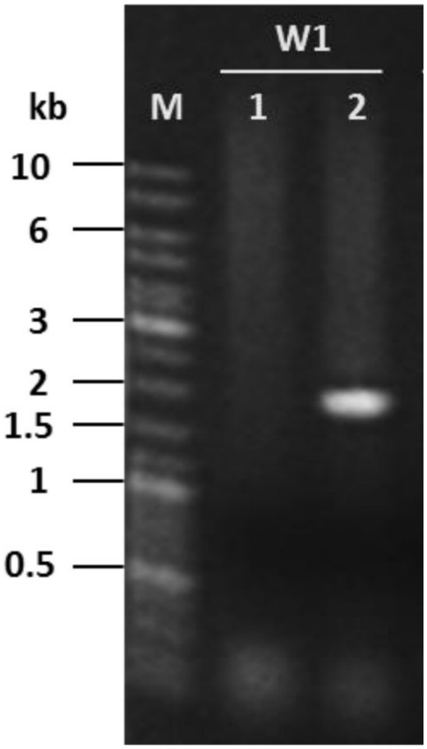

Fig. S1 Amplified PCR products from genomic DNA of *B. amyloliquefaciens* subsp. *plantarum* W1 by primers specific for *amy*A and *amy*E genes. Lane M = 2-log DNA ladder (Thermo Scientific, USA), Lane 1 = primer-*amy*A, and Lane 2 = primer-*amy*E

Table S2 Remaining BTEX (%) and biodegradation (%) in a liquid medium system containing live and dead cells of *B. amyloliquefaciens* strain W1 after 24 h (BTEX was supplemented as a single substrate.)

Substrate	Remaining BTEX (%)		Biodegradation (%)
	Dead cells	Live cells	
Benzene	97±3.2	68±0.5	29±2.7
Toluene	98±1.6	64±5.5	34±7.1
Ethylbenzene	83±0.5	54±1.9	29±1.4
p-Xylene	78±1.0	49±1.6	30±0.6
m-Xylene	81±1.0	63±6.0	18±5.0
o-Xylene	88±1.3	74±2.7	14±4.0

Table S3 Remaining BTEX (%) in a liquid medium system containing *B. amyloliquefaciens* strain W1 after 0.5, 1 and 7 days (BTEX was supplemented as a single substrate or a mixture.)

Substrate	12 h		1 day		7 days	
	Single	Mixture	Single	Mixture	Single	Mixture
Benzene	72±1.8	87±0.3	68±0.5	72±2.6	50±5.3	63±1.9
Toluene	71±4.9	84±4.2	64±5.5	59±6.2	49±3.3	51±0.4
Ethylbenzene	69±3.5	78±5.5	54±1.9	52±3.3	39±1.3	47±2.5
p-Xylene	70±4.1	75±9.2	49±1.6	50±2.3	32±2.1	35±2.0
m-Xylene	72±5.2	78±5.7	63±6.0	51±1.9	43±1.8	43±2.6
o-Xylene	73±5.7	82±8.6	74±2.7	59±2.1	53±5.6	57±4.4

Table S4 First-order kinetics of BTEX biodegradation in a liquid medium system and a soil slurry system containing *B. amyloliquefaciens* strain W1 (BTEX was supplemented as a single substrate or a mixture.)

Substrate	Liquid medium system				Soil slurry system	
	Single		Mixture		Mixture	
	k (1/h)	$t_{1/2}$ (h)	k (1/h)	$t_{1/2}$ (h)	k (1/h)	$t_{1/2}$ (h)
Benzene	0.016	43.1	0.014	52.0	0.006	121.3
Toluene	0.019	38.9	0.022	32.8	0.006	127.3
Ethylbenzene	0.026	27.1	0.028	25.4	0.005	146.0
p-Xylene	0.030	23.2	0.029	23.9	0.006	111.2
m-Xylene	0.019	37.4	0.028	25.1	0.005	128.7
o-Xylene	0.014	48.3	0.022	31.7	0.004	160.3

Table S5 Remaining BTEX (%) in a liquid medium and soil slurry system containing *B. amyloliquefaciens* strain W1 after 1, 7 and 30 days (BTEX was supplemented as a BTEX mixture.)

Substrate	1 day		7 days		30 days	
	Liquid medium	Soil slurry	Liquid medium	Soil slurry	Liquid medium	Soil slurry
Benzene	76±7.9	95±4.5	66±1.4	79±6.0	27±4.2	49±1.0
Toluene	73±4.5	97±3.7	56±1.5	72±5.0	13±2.0	29±3.8
Ethylbenzene	66±3.4	99±2.5	49±3.4	74±6.0	10±1.9	32±0.4
p-Xylene	63±3.0	95±4.9	36±3.1	59±5.3	3±0.6	14±0.6
m-Xylene	66±2.5	96±3.9	45±3.2	69±6.0	7±1.4	24±1.0
o-Xylene	73±3.0	97±3.6	60±2.0	79±6.4	19±3.4	46±3.0

Table S6 Mass spectra of metabolites detected during biodegradation of benzene

No.	MS/MS pattern
1.	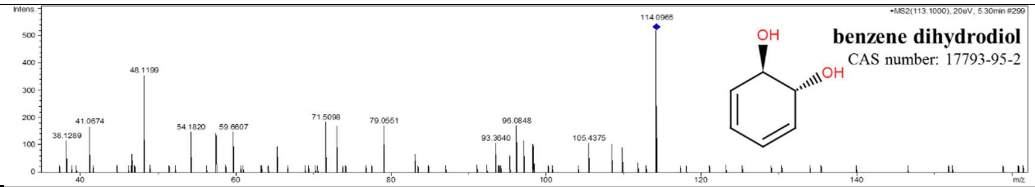
2.	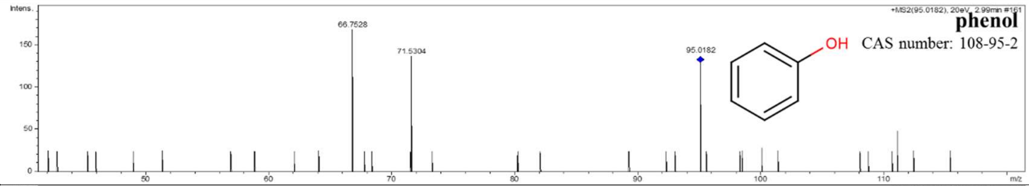
3.	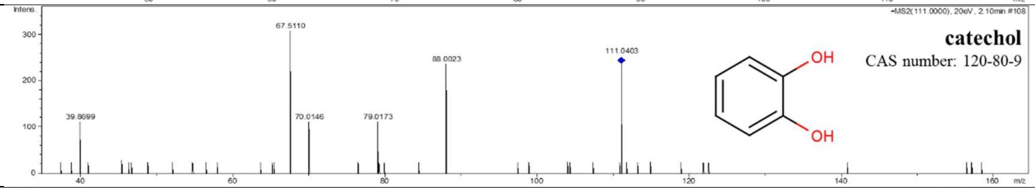
4.	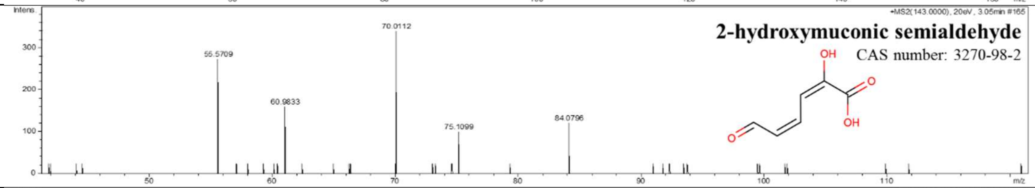
5.	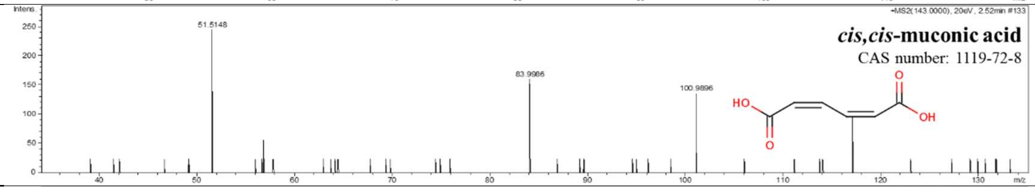
6.	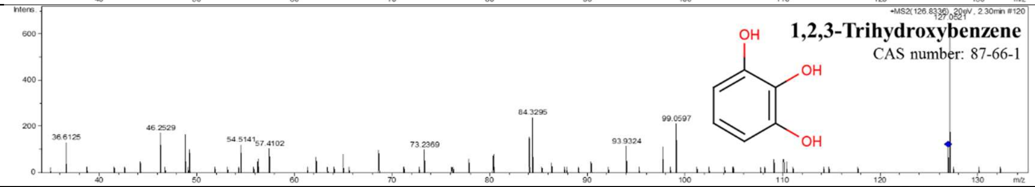
7.	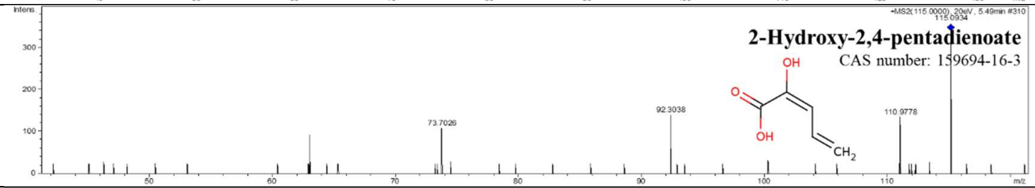
8.	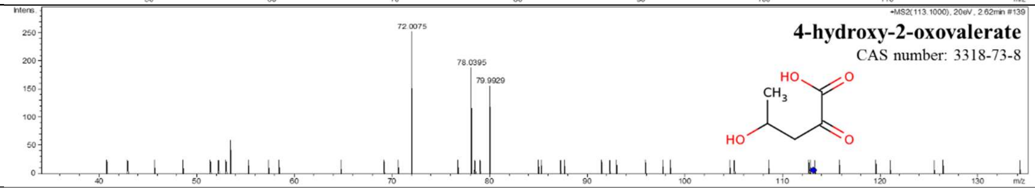

Table S7 Mass spectra of metabolites detected during biodegradation of toluene

No.	MS/MS pattern
1.	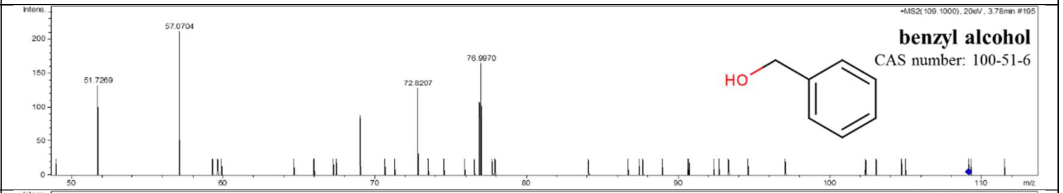
2.	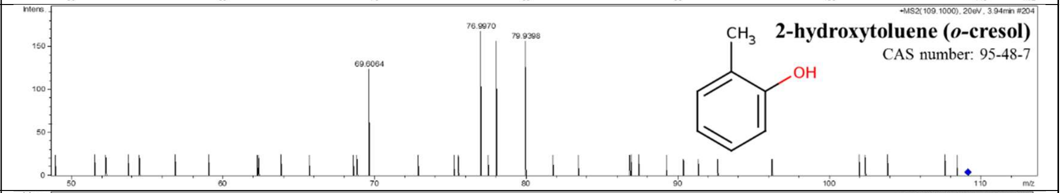
3.	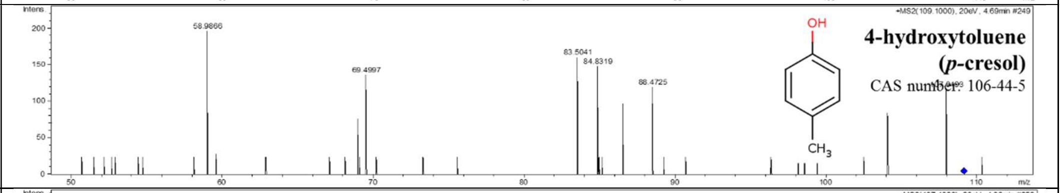
4.	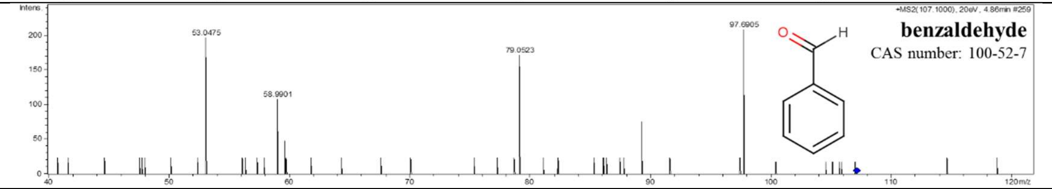
5.	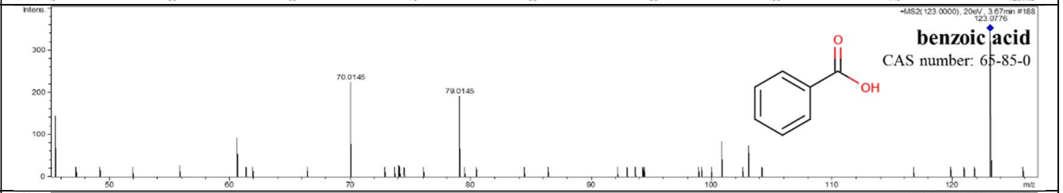
6.	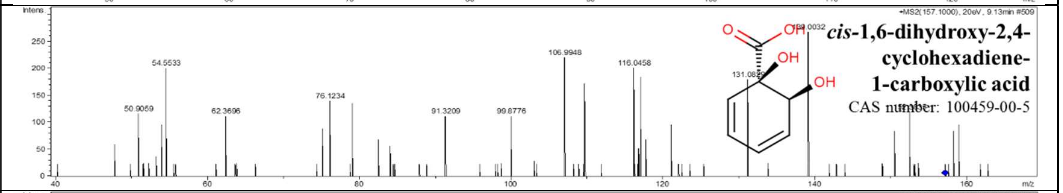
7.	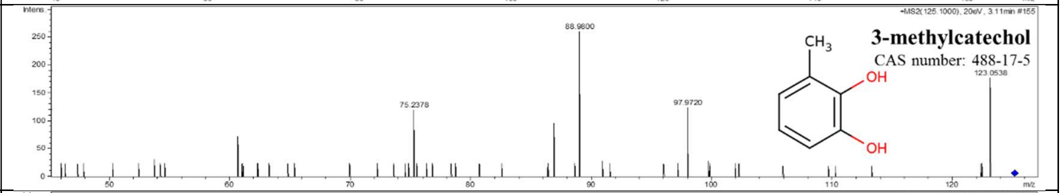
8.	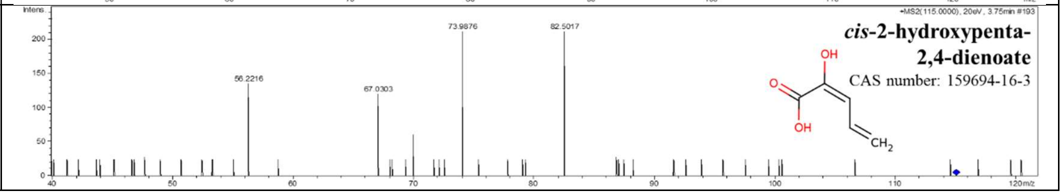

Table S7 (continued) Mass spectra of metabolites detected during biodegradation of toluene

No.	MS/MS pattern
9.	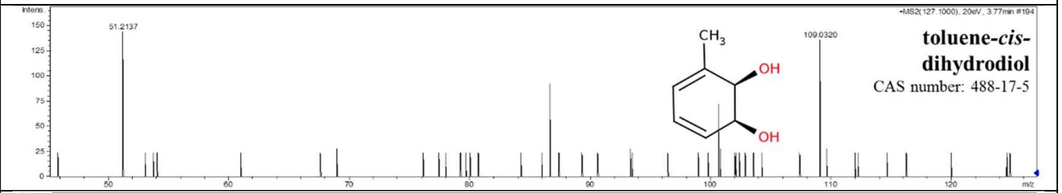
10.	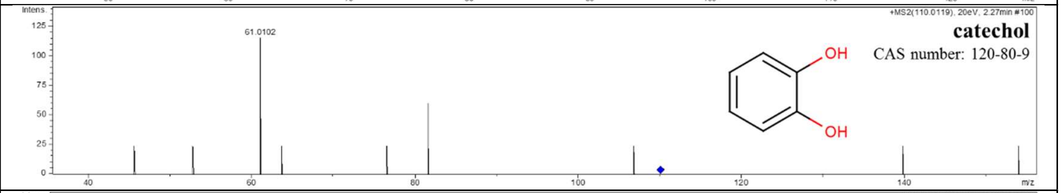
11.	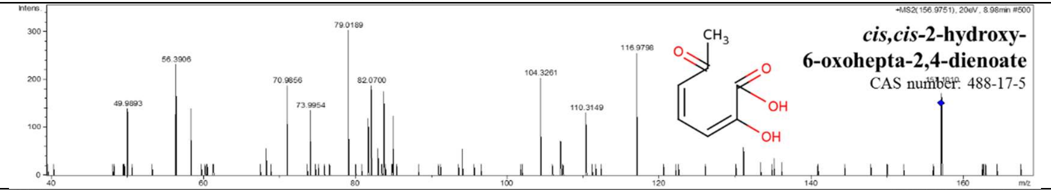
12.	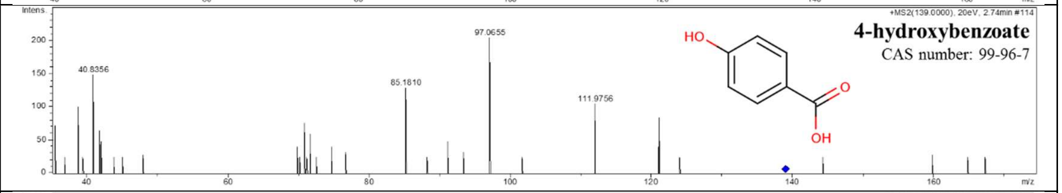
13.	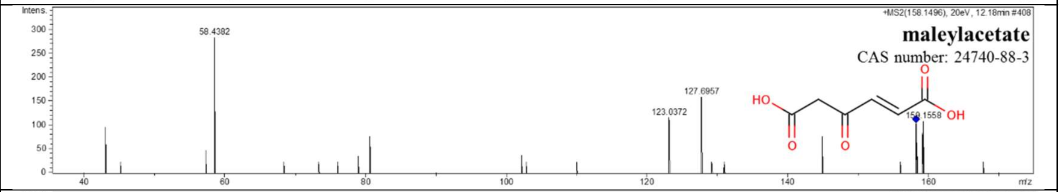
14.	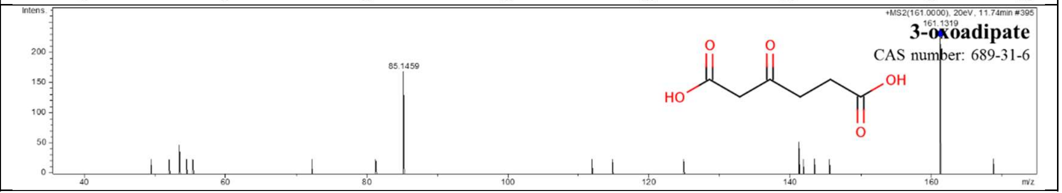

Table S8 Mass spectra of metabolites detected during biodegradation of ethylbenzene

No.	MS/MS pattern
1.	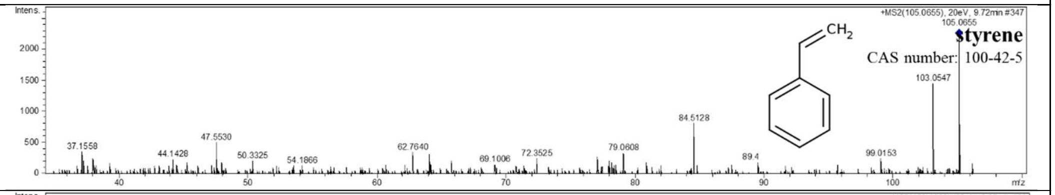
2.	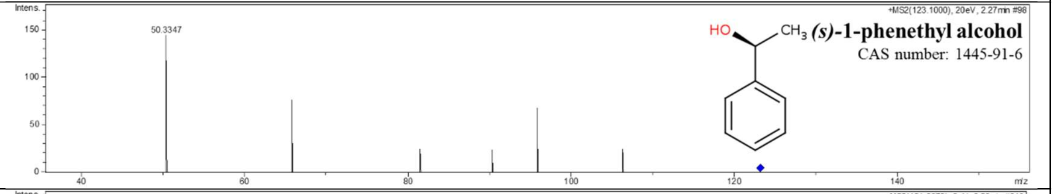
3.	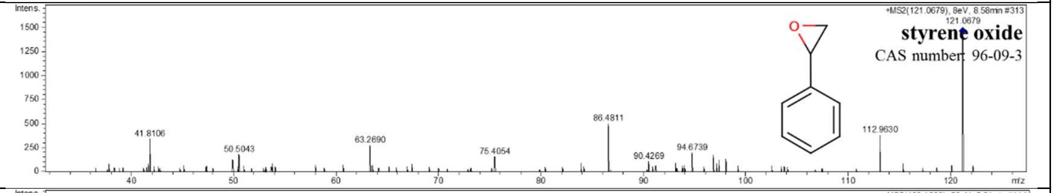
4.	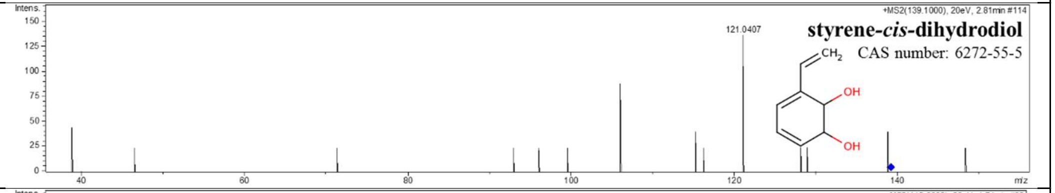
5.	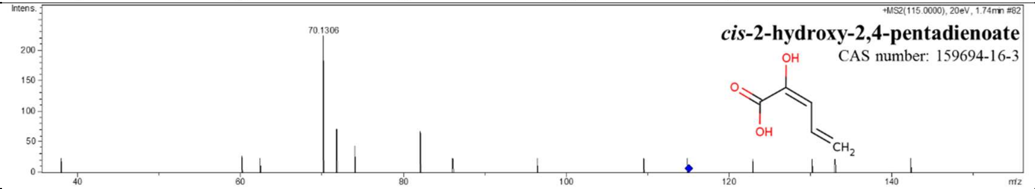
6.	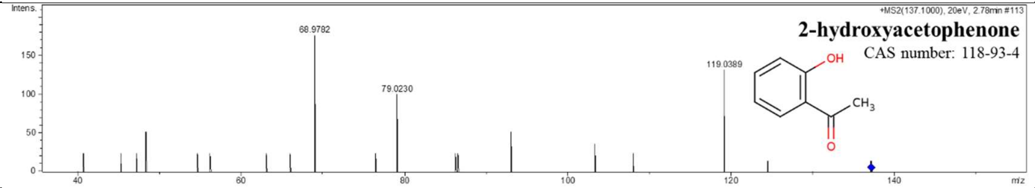
7.	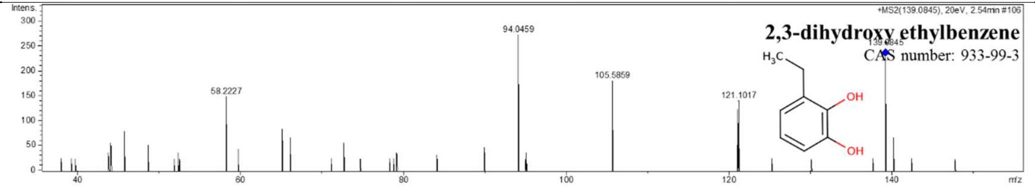
8.	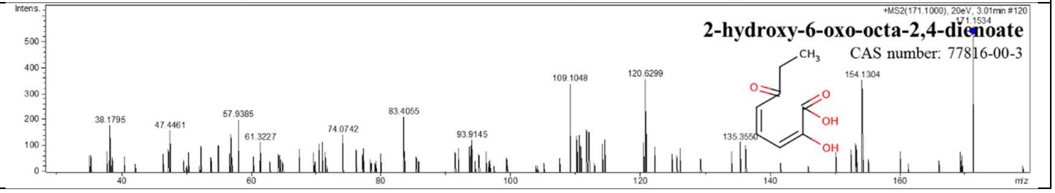

Table S9 Mass spectra of metabolites detected during biodegradation of *o*-xylene

No.	MS/MS pattern
1.	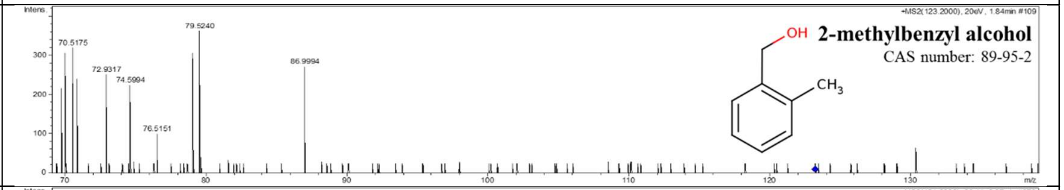
2.	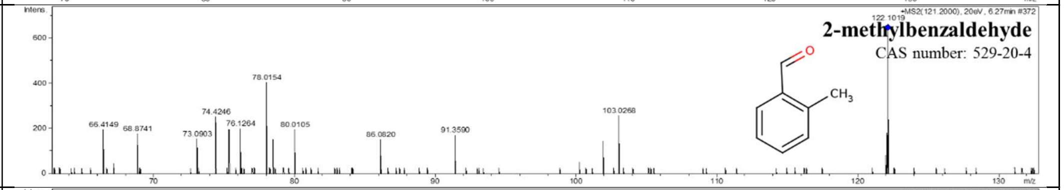
3.	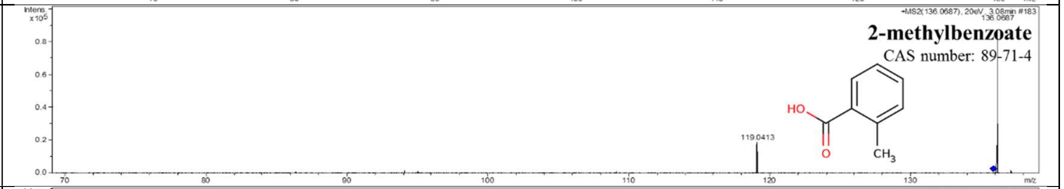
4.	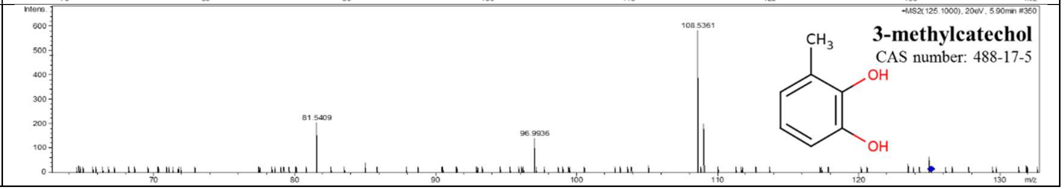

Table S10 Mass spectra of metabolites detected during biodegradation of *m*-xylene

No.	MS/MS pattern
1.	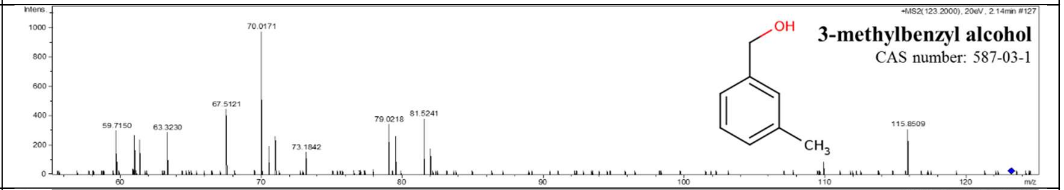
2.	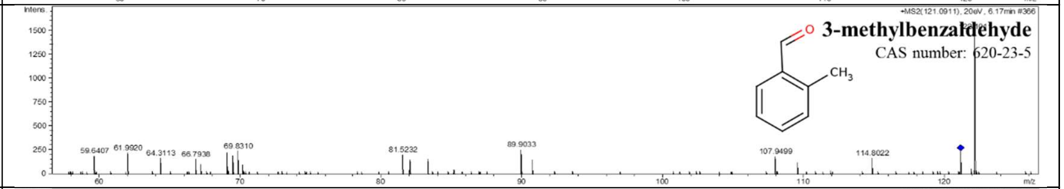
3.	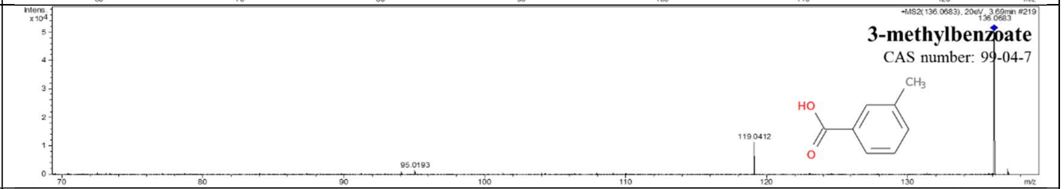
4.	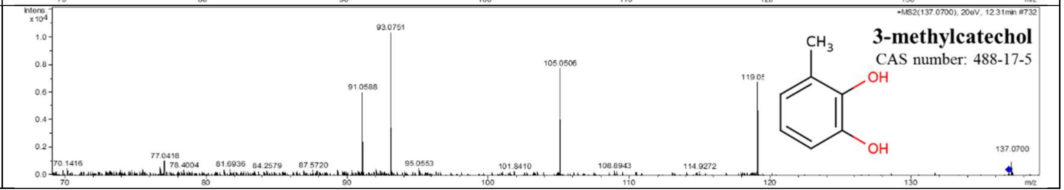

Table S11 Mass spectra of metabolites detected during biodegradation of *p*-xylene

No.	MS/MS pattern
1.	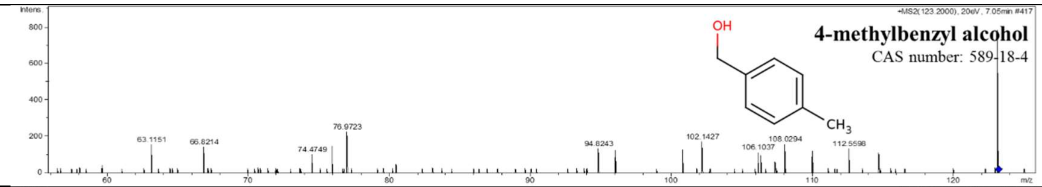
2.	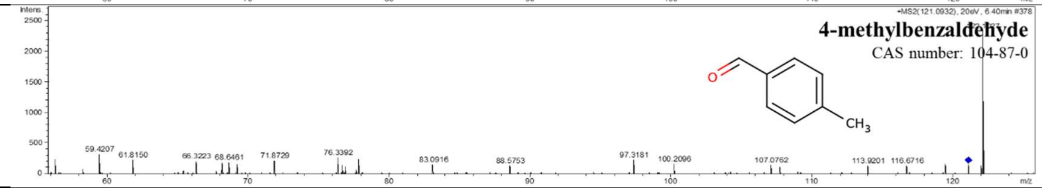
3.	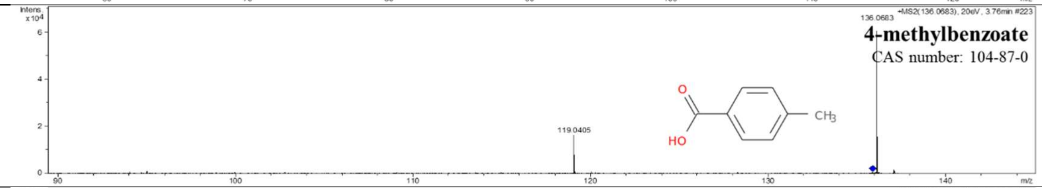
4.	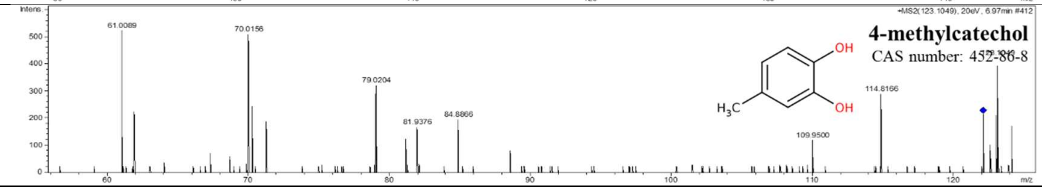

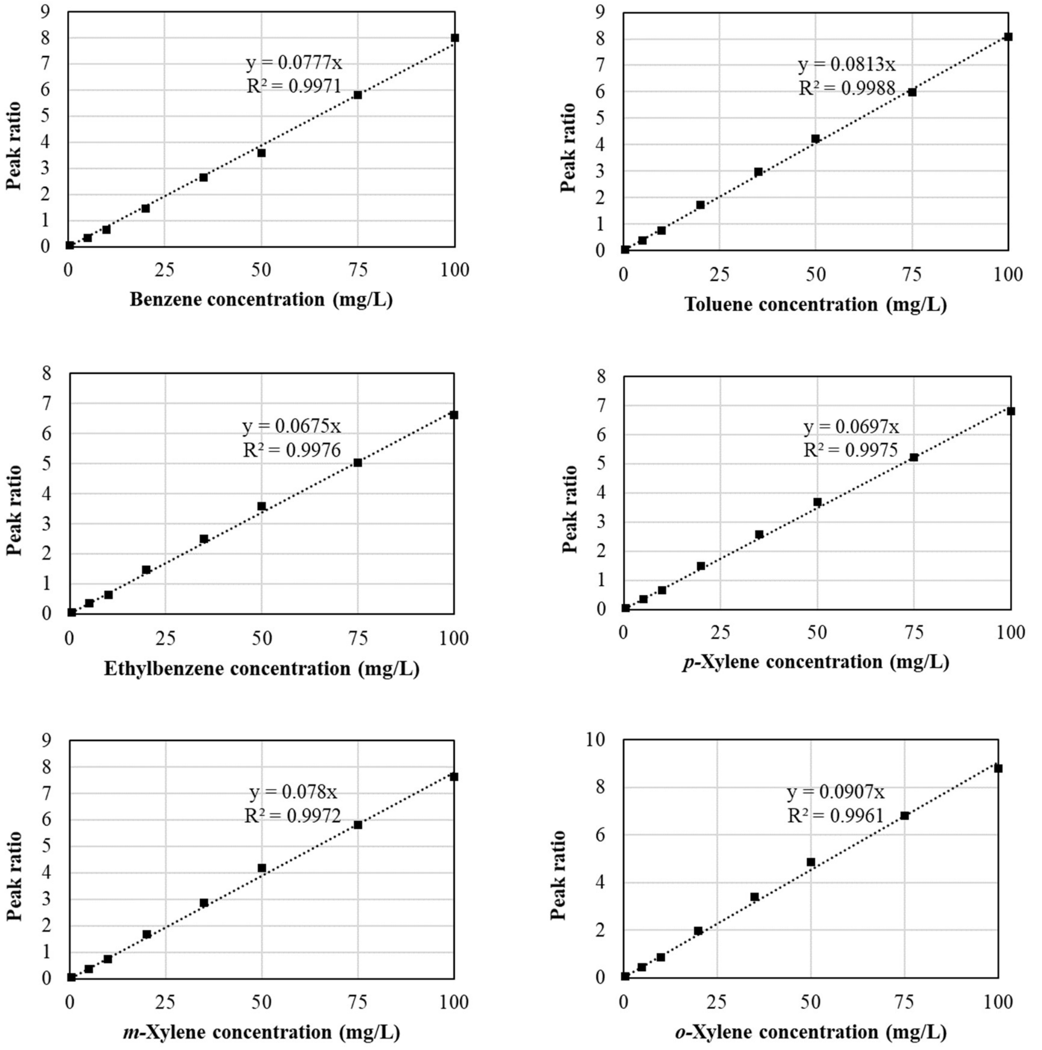

Fig. S2 Standard curves of BTEX analyzed by GC-FID

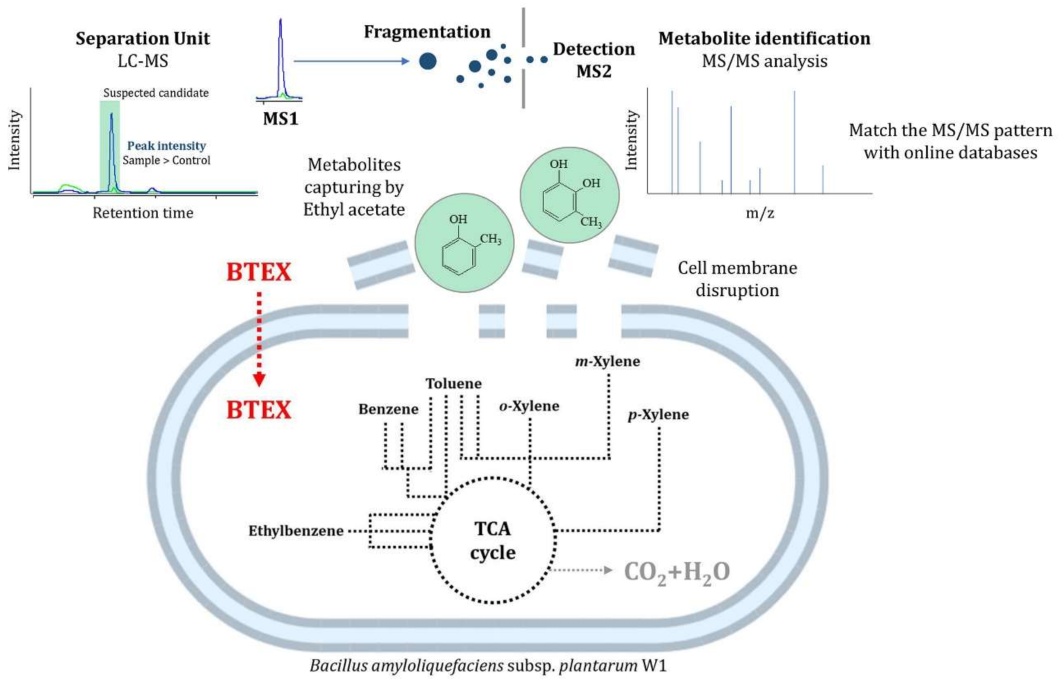

Fig. S3 Metabolites tracking protocol used in this study
